# Clinical Considerations and Outcomes for Spine Surgery Patients with a History of Transplant: A Systematic Scoping Review Protocol

**DOI:** 10.3390/mps5030047

**Published:** 2022-06-05

**Authors:** Roshini Kalagara, Zerubabbel K. Asfaw, Matthew T. Carr, Addison Quinones, Lisa Genadry, Zaid Nakadar, Anzila Haris, Alexander J. Schupper, Jonathan S. Gal, Tanvir F. Choudhri

**Affiliations:** 1 Department of Neurosurgery, Icahn School of Medicine at Mount Sinai, New York, NY 10029, USA; zerubabbel.asfaw@icahn.mssm.edu (Z.K.A.); matthew.carr@mountsinai.org (M.T.C.); addison.quinones@icahn.mssm.edu (A.Q.); lisa.genadry2@mountsinai.org (L.G.); alex.schupper@gmail.com (A.J.S.); tanvir.choudhri@mountsinai.org (T.F.C.); 2 Department of Medical Education, College of Medicine, SUNY Downstate Health Sciences University, Brooklyn, NY 11203, USA; zaid.nakadar@downstate.edu; 3 College of Human Ecology, Cornell University, Ithaca, NY 14850, USA; ah692@cornell.edu; 4 Department of Anesthesiology, Perioperative & Pain Medicine, Icahn School of Medicine at Mount Sinai, New York, NY 10029, USA; jonathan.gal@mountsinai.org

**Keywords:** transplant, spine surgery, patient outcomes

## Abstract

Spine surgery patients with a history of organ transplantation are a complex population due to their unique anesthetic considerations, immunologic profiles, drug interactions, and potential organ dysfunction. It is common for these patients to develop neck/back pain and pathology that warrants surgical intervention. However, there is a relative dearth of literature examining their outcomes and clinical considerations. The purpose of this protocol is to investigate their clinical outcomes following spine surgery and medical management. We perform a systematic literature search using PRISMA-ScR guidelines to include case reports, cohort studies, and retrospective analyses. The search terms include kidney, liver, heart, pancreas, lung, and bone marrow for transplants of interest and contain an extensive list of terms covering spine surgery. The search is conducted using Medline, Embase, and the Cochrane Central Register of Controlled Trials. A thorough examination of titles and abstracts is performed followed by data extraction. The data points include patient demographics, past medical history, spine procedural information, and clinical outcomes. This systematic review will aid clinicians in identifying demographics, medical management, and clinical outcomes for spine surgery patients with a previous organ transplant. These findings will highlight the gaps in the knowledge of this complex population and stimulate further research.

## 1. Background

Organ transplantation, a prevalent and viable therapy for end-stage organ failures, has become increasingly common in recent years [1]. The second decade of the 21st century was marked by a record-breaking streak of organ transplant operations in the United States (US) [2]. Advancements in organ preservation methods, immunosuppressive therapies that enhance graft longevity, general post-transplant care, and long-term management have significantly improved outcomes. As a result of such innovations, the ever-increasing population of post-transplant patients has a concurrently improving life expectancy [3,4]. More of these patients have become eligible to undergo elective surgeries that address disorders that may not be directly related to their history of transplant. 

Among the US population, spine surgery remains common and is expected to increase in demand in the coming decades [5,6]. The expected rise in spine surgery operations coupled with the increased longevity of post-transplant patients is likely to result in a great increase in transplant patients undergoing spine surgery. Spine surgery outcomes in patients who have received organ transplant remain largely unstudied despite long-term data demonstrating that neck/back pain and spinal pathology are common within the population of individuals who have received transplant surgery [7,8]. Previous literature that focuses on spine surgery patients with a history of organ transplant has largely been limited to case reports and case series.

The purpose of this scoping review is to analyze the clinical considerations, clinical outcome/postoperative variables, and quality of previous studies which examine the patient population that has received organ transplant prior to spine surgery.

## 2. Methods

The scope of this review will consider studies focusing on spine surgery in patients with a history of transplant. Studies that report on transplant patients with a history of spine surgery will not be considered in the review. The following type of transplants will be included in the review: heart, lung, liver, kidney, pancreas, pancreatic islet, and bone marrow. Studies that focus on bone grafts, vascularized composite allotransplants, and other types of transplants will not be included. This review will also not consider studies on neurosurgery patients with a transplant history who undergo a cerebral procedure.

### 2.1. Study Selection

#### 2.1.1. Study Design

This review will consider all empirical and quantitative studies for inclusion. The included studies must report the clinical considerations that need to be accounted for when a spine surgery patient has a history of transplant, such as immunosuppression use and medical comorbidities. Moreover, the included studies should also present the clinical outcomes of the patient population of interest. Only single case studies and articles published in English will be included. This systematic scoping review follows PRISMA-ScR guidelines [9,10].

#### 2.1.2. Participants

This scoping review will include studies that involve spine surgery patients with a history of organ and bone marrow transplant. Study participants may be of any age as long as they received a transplant before undergoing spine surgery. Studies on animal models will not be included.

### 2.2. Search Strategy

Searches will be conducted to locate studies published analyzing transplant patients who subsequently underwent spine surgery. We will include language that includes bone marrow and various solid organ transplants, such as the liver, heart, and lung. Moreover, we will utilize broad language detailing spinal procedures to capture the intended patient cohort, including search terms such as laminoplasty and decompression surgery. With this combination of search terms, we hope to capture a broad range of studies for our review (Supplementary Materials, Table S1). We will synthesize searches from three databases: Medline, Embase, and the Cochrane Central Register of Controlled Trials. All searches were performed in July 2021. 

### 2.3. Screening

**Figure 1:**
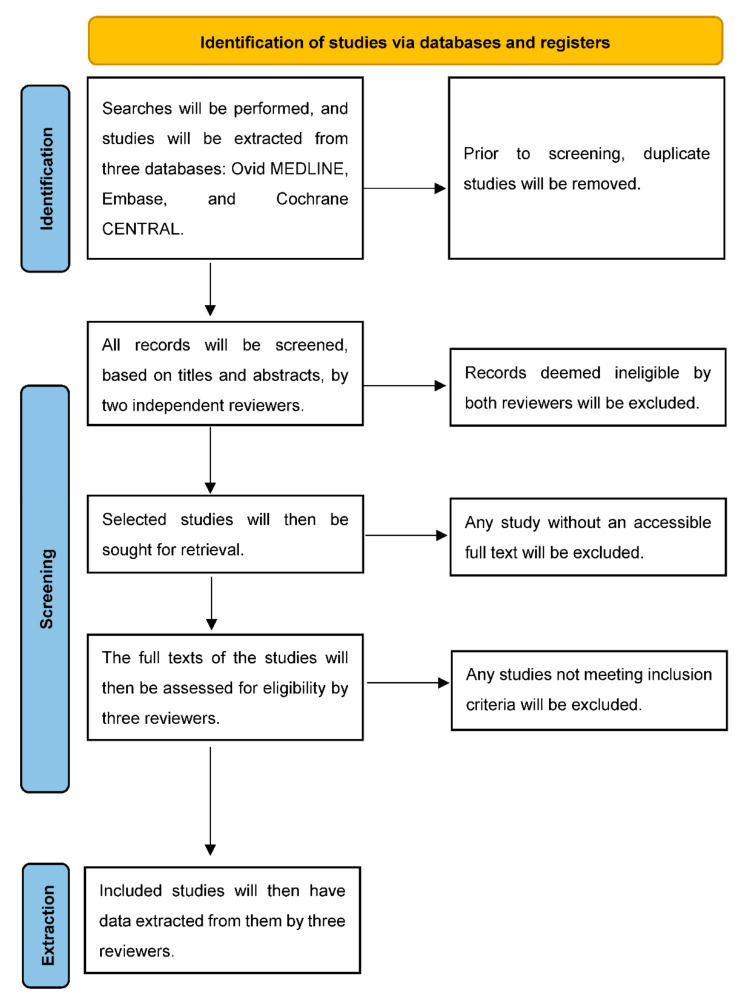
Diagram of study screening and selection.

Studies will be uploaded into Covidence for review and duplicates will be removed. Two reviewers will independently perform title and abstract screening of all studies and conflicts will be adjudicated. A full text review will be performed by three reviewers to ensure inclusion in the study. Any questions during this process will be submitted to an additional supervising reviewer. All studies on spine surgery patients with a history of the transplant types specified above will be eligible for inclusion, regardless of setting, as long as the temporal arrangement of surgeries aligns with the inclusion criteria. The study selection and screening procedures are detailed in Figure 1.

### 2.4. Data Extraction

Data extraction will be performed by three reviewers, and any questions or conflicts will be discussed and adjudicated with three additional reviewers. The data extraction tool will be geared to collect information concerning the transplant procedure, the spinal surgery, and the overall outcomes. Moreover, demographic information about the patients, including medications, immunosuppression, and other comorbidities will be extracted from the included studies. 

### 2.5. Data Presentation

The data collected will then be analyzed and prepared for presentation. Qualitative data, including patient demographics and surgical variables, will be presented in a tabular format in the scoping review paper. Quantitative analyses will be performed on the outcome variables and also presented in the scoping review. Gaps in the literature will be addressed, and recommendations for future work will also be discussed.

## 3. Discussion

Organ transplantation is a valuable therapeutic strategy for patients presenting with end stage organ failure. As new therapies and management practices are implemented, it is expected that these patients will have an improved survival. With this improved survival, it is expected that these patients will develop further maladies such as spinal pathologies requiring surgical intervention [8]. There is a relative lack of research on the topic of post-transplant patients who subsequently go on to require spine surgery. To the best of our knowledge, this review will serve as the first systematic scoping review examining the treatment and outcomes of this patient population. In the realm of spine surgery, there is a need for this type of systematic review to collate the sparse existing knowledge.

The primary objective of this review is to collect the relevant evidence regarding the pathology, medical management, and clinical outcomes of post-transplant spine surgery patients. This review will attempt to identify trends in this patient population that may prove beneficial for improvement in their care. This review has the potential to elicit a dichotomy of clinical outcomes between post-transplant spine surgery patients and the general spine surgery population. Differences may be found involving mortality, infection rate, re-admission rate, vertebral location, length of hospital stay, etc. Additionally, this study may help to identify which types of transplants are at most risk for poorer outcomes.

During this analysis, a few challenges are anticipated that may hinder this review. It may be hard to draw definitive conclusions about this patient population if the literature mainly consists of individual case reports or large retrospective analyses lacking granular data. If the opportunity presents itself to pool data of the same organ or same pathology, then this will be the optimal approach. Another limitation is that there may be a large range of heterogeneity in the literature in terms of treatment and reporting, as there are not well-established guidelines for this patient population. 

Overall, the culmination of this data will also help to highlight gaps in the understanding of these patients which will fuel further research. It is the intention that this study will eventually lead to the incorporation of transplant factors into risk modeling for post-transplant spine surgery patients, which will increase the efficacy of decision making for clinicians, ultimately resulting in greater outcomes for these patients. Moreover, by creating a framework for studying this medically complex population, this study will be able to serve as a baseline for understanding how these clinical factors affect surgical outcomes and strategies to optimize care utilization.

## Data Availability

Not applicable.

## Supplementary Materials

The following supporting information can be downloaded at: https://www.mdpi.com/article/10.3390/mps5030047/s1, Table S1: Search Criteria.
