# Supplementary material for: Clinical Considerations and Outcomes for Spine Surgery Patients with a History of Transplant: A Systematic Scoping Review Protocol
Source: Methods Protoc. 2022 Jun 5;5(3):47. doi: 10.3390/mps5030047 (PMC9229861; doi:10.3390/mps5030047)
Supplement: Supplementary file 1 [file mps-05-00047-s001.zip › mps-1644958-supplementary.pdf]

methods and protocols

MDPI

Supplemental Table S1. Search Criteria

Ovid MEDLINE® and Epub Ahead of Print, In-Process, In-Data-Review and Other Non-Indexed Citations, Daily and Versions® 1946 to 29 July 2021—323 Results

1. exp Organ Transplantation/
2. exp bone marrow transplantation/
3. ((Organ or heart or liver or kidney or pancreas or heart-lung or bone marrow) adj3 (transplant* or graft*)).mp.
4. or/1-3
5. Lumbar Vertebrae/su [Surgery]
6. Spine/su [Surgery]
7. Cervical Vertebrae/su [Surgery]
8. Thoracic Vertebrae/su [Surgery]
9. Intervertebral Disc/su [Surgery]
10. exp Decompression, Surgical/
11. Laminoplasty/
12. exp Arthrodesis/
13. ((Spine or spinal or vertebra* or intervertebral disc* or intervertebral disk*) adj3 surg*).mp.
14. (Diskectom* or discectom*).mp.
15. Laminectom*.mp.
16. Laminoplast*.mp.
17. Surgical decompression*.mp.
18. decompression surg*.mp.
19. Arthrodes*.mp.
20. ((Spine or spinal) adj fusion*).mp.
21. or/5-20
22. 4 and 21
23. (animal*.hw. or exp animal/) not human/
24. 22 not 23

Embase Classic and Embase 1947 to 29 July 2021—786 results

1. exp Organ Transplantation/
2. bone marrow transplantation/
3. ((Organ or heart or liver or kidney or pancreas or heart-lung or bone marrow) adj3 (transplant* or graft*)).mp.
4. or/1-3
5. lumbar vertebra/su [Surgery]
6. spine/su [Surgery]
7. intervertebral disk/su [Surgery]
8. exp decompression surgery/
9. Laminoplasty/
10. exp Arthrodesis/
11. exp discectomy/
12. ((Spine or spinal or vertebra* or intervertebral disc* or intervertebral disk*) adj3 surg*).mp.
13. (Diskectom* or discectom*).mp.
14. Laminectom*.mp.
15. Laminoplast*.mp.
16. Surgical decompression*.mp.
17. decompression surg*.mp.
18. Arthrodes*.mp.
19. ((Spine or spinal) adj fusion*).mp.
20. or/5-19
21. 4 and 20
22. (animal*.hw. or exp animal/) not human/
23. 21 not 22

Cochrane CENTRAL—609 results

1. MeSH descriptor: [Organ Transplantation] explode all trees
2. MeSH descriptor: [Bone Marrow Transplantation] explode all trees
3. ((organ OR heart OR liver OR kidney OR pancreas OR heart-lung OR bone marrow) NEAR/3 (transplant* OR graft*)):ti,ab,kw
4. #1 OR #2 OR #3
5. MeSH descriptor: [Lumbar Vertebrae] this term only and with qualifier(s): [surgery - SU]
6. MeSH descriptor: [Spine] this term only and with qualifier(s): [surgery - SU]
7. MeSH descriptor: [Cervical Vertebrae] this term only and with qualifier(s): [surgery - SU]
8. MeSH descriptor: [Thoracic Vertebrae] this term only and with qualifier(s): [surgery - SU]
9. MeSH descriptor: [Intervertebral Disc] this term only and with qualifier(s): [surgery - SU]
10. MeSH descriptor: [Decompression, Surgical] explode all trees
11. MeSH descriptor: [Laminoplasty] this term only
12. MeSH descriptor: [Arthrodesis] explode all trees
13. ((Spine OR spinal OR vertebra* OR intervertebral disc* OR intervertebral disk*) NEAR/3 surg*):ti,ab,kw
14. (Diskectom* OR discectom*):ti,ab,kw
15. (Laminectom*):ti,ab,kw
16. (Laminoplast*):ti,ab,kw
17. (Surgical decompression*):ti,ab,kw
18. (decompression surg*):ti,ab,kw
19. (Arthrodes*):ti,ab,kw
20. ((Spine OR spinal) NEAR fusion*):ti,ab,kw
21. {OR #5-#20}
22. #4 AND #21 in Trials
